# Control of Invasive *Salmonella* Disease in Africa: Is There a Role for Human Challenge Models?

**DOI:** 10.1093/cid/civ673

**Published:** 2015-10-07

**Authors:** Malick M. Gibani, Celina Jin, Thomas C. Darton, Andrew J. Pollard

**Affiliations:** Oxford Vaccine Group, Department of Paediatrics, University of Oxford and the National Institute for Health Research Oxford Biomedical Research Centre, United Kingdom

**Keywords:** human challenge study, controlled human infection study, typhoid fever, *Salmonella* Typhi, invasive nontyphoidal *Salmonella*

## Abstract

Invasive *Salmonella* disease in Africa is a major public health concern. With evidence of the transcontinental spread of the *Salmonella* Typhi H58 haplotype, improved estimates of the burden of infection and understanding of the complex interplay of factors affecting disease transmission are needed to assist with efforts aimed at disease control. In addition to *Salmonella* Typhi, invasive nontyphoidal *Salmonella* are increasingly recognized as an important cause of febrile illness and mortality in sub-Saharan Africa. Human experimental oral challenge studies with *Salmonella* can be used as a model to offer unique insights into host–pathogen interactions as well as a platform to efficiently test new diagnostic and vaccine candidates. In this article, we review the background and use of human challenge studies to date and discuss how findings from these studies may lead to progress in the control of invasive *Salmonella* disease in Africa.

There is a pressing need to systematically address the problem of invasive *Salmonella* disease in Africa [1, 2]; however, current epidemiological evidence suggests a complex and dynamic situation across the continent. The few accurate prevalence data for *Salmonella enterica* subspecies *enterica* serovars Typhi and Paratyphi, responsible for causing enteric fever, demonstrate a preponderance of *Salmonella* Typhi with marked geographic variation and urban localization [3]. In some urban slums areas, for example, rates seen are comparable to those in Southeast Asia [4]. Recent events include the rapid dominance of an emerging *Salmonella* Typhi haplotype, termed H58, associated with multidrug resistance and occasional reduced fluoroquinolone susceptibility [4–7]. Recent phylogeographical analysis has demonstrated an increasing incidence of *Salmonella* Typhi H58 across Africa over the past 30 years, following multiple transmission events from Asia. The introduction of H58 appears to be associated with clonal replacement of resident non-H58 antibiotic-susceptible strains, resulting in transformation of the *Salmonella* Typhi epidemiology across Africa and the occurrence of multidrug-resistant outbreaks in regions where the disease was previously underappreciated [7].

In Africa, the problem of invasive *Salmonella* disease is exacerbated by high rates of invasive nontyphoidal *Salmonella* (iNTS) infection, caused by serovars of *Salmonella enterica* other than Typhi or Paratyphi. In this setting, the estimated annual incidence of iNTS disease approaches 227 per 100 000 population [8], and recognized susceptibility factors include younger age (6 months to 3 years), malnutrition, and comorbid malaria or human immunodeficiency virus infection [2]. NTS strains most commonly isolated from bacteremic, febrile patients vary by location but frequently include *Salmonella* Typhimurium and *Salmonella* Enteritidis [9]. Sequencing of isolates from across sub-Saharan Africa has identified *Salmonella* Typhimurium multilocus sequence type 313 (ST313) as the major cause of invasive disease in the region. Further whole-genome sequencing has identified interesting converging evolutionary changes by the ST313 strain toward a genotype resembling the classic enteric fever-causing *Salmonella* serovars. These changes are characterized by degradation of the genomic repertoire by multiple gene deletions and pseudogene formation. This suggests that ST313 may have undergone recent host adaptation to a human niche, as has *Salmonella* Typhi sometime in the last few thousand years [10, 11].

Further understanding of the host–pathogen dynamics that occur during invasive *Salmonella* disease is required to recognize factors affecting disease transmission and human immune responses to infection. Recently, a program of typhoid and paratyphoid human challenge studies has been undertaken by the Oxford Vaccine Group at the University of Oxford based on previous typhoid challenge work performed by the University of Maryland School of Medicine. These studies aim to address several critical knowledge gaps, thereby informing disease transmission models and accelerating the development of public health improvement strategies. In this article, we review the background and use of human challenge studies to date and discuss how findings from these studies can offer insights to assist with the control of typhoidal *Salmonella* disease. We also discuss the feasibility and complexities of developing a human challenge study of NTS disease, and how such a model could help to inform public health strategies to control iNTS disease in Africa.

## DEVELOPMENT OF HUMAN TYPHOID CHALLENGE STUDIES

A systematic program of *Salmonella* Typhi challenge studies was undertaken in Maryland between 1952 and 1974. Through meticulous collection of clinical and microbiological data from almost 2000 participants, many new advances were made in the understanding of typhoid infection pathogenesis and human responses to infection [12, 13]. These studies also demonstrated the safety and utility of human challenge models in vaccine evaluation, as exemplified by assessment of oral Ty21a vaccination [14, 15].

In 2011, the Oxford Vaccine Group at the University of Oxford reestablished a *Salmonella* Typhi human challenge model with the specific aim of creating a platform to assess novel vaccine candidates [16]. The model developed uses an outpatient, ambulatory design selecting healthy adults aged 18–60 years who undergo a rigorous screening process before enrollment [16]. Although the ethical and design considerations are beyond the scope of this review, they have been summarized elsewhere [17–19]. To date, >160 participants have been safely challenged in the Oxford studies.

Oral challenge is performed using a defined dose of *Salmonella* Typhi suspended in sodium bicarbonate solution. Following challenge, participants are reviewed daily for 14 days, with regular assessments made of clinical and symptomatic features, routine biochemical/hematological measurements, and daily blood and stool cultures. A diagnosis of enteric fever requires participants to meet predefined criteria of fever ≥38°C lasting ≥12 hours and/or *Salmonella* Typhi bacteremia, after which a 2-week course of oral antibiotics is commenced. Participants who reach the 14th day of challenge without meeting diagnostic criteria are also treated with antibiotics [16]. The highly intensive and controlled nature of these types of studies enables the longitudinal tracking of a disease process from a defined point of exposure through to infection, diagnosis, and treatment, allowing observations and measurements to be made that would be difficult to obtain in natural infection.

### Infectious Dose

The infectious dose of *Salmonella* bacteria required to induce clinical illness is an important factor to understand when assessing disease transmission and control methods [20, 21]. In the initial typhoid challenge study performed at Oxford, a predetermined dose escalation strategy was followed, starting with a dose of 10^3^ colony-forming units (CFU), increasing in log-fold increments, until an attack rate of 60%–75% was achieved [16]. An attack rate of 55% was observed with a challenge dose of 10^3^ CFU, rising to 65% at 10^4^ CFU [16]. Although the same *Salmonella* Typhi Quailes strain was used in both the Oxford and Maryland studies, the coadministration of sodium bicarbonate buffer immediately prior to challenge in the Oxford study resulted in a 4-log_10_ lower dose of *Salmonella* Typhi required to produce an attack rate of 65% [22]. Whereas lower levels of pathogen exposure are thought to more accurately reflect natural infection with *Salmonella* Typhi, differences in attack rate observed between the Oxford and Maryland studies may in part be attributable to methodical factors including the definition of enteric fever used [16, 23], method of challenge agent preparation [22], and use of bicarbonate buffer [16], as well as preexisting immunity of study volunteers.

### Blood Culture Surveillance

In the Oxford study, daily blood cultures were taken from all participants from 12 hours after challenge until day 14 or 96 hours postdiagnosis. A minority of participants (3/24 [12.5%]) were diagnosed with enteric fever based upon clinical criteria alone (fever ≥38°C lasting ≥12 hours) without evidence of bacteremia. This finding highlights the relatively low sensitivity of automated blood culture even with repeated sampling in a high-income setting, supporting the view that reliance on blood culture alone may underestimate the true disease burden in African settings where multiple alternative causes for fever exist.

Conversely, 4 of 24 diagnosed participants (16.6%) in our model had detectable *Salmonella* Typhi bacteremia without clinical signs of enteric fever. Two of these 4 participants had a significant increase in anti-lipopolysaccharide (LPS) antibody from a low baseline titer, suggesting that the humoral immune system was exposed to the bacteria at some point after challenge (unpublished observations). Subclinical or asymptomatic infection has long been suspected to occur following exposure to *Salmonella* Typhi and may play an important role in onward disease transmission. Human challenge studies permit the detailed assessment of this subset of people, who are difficult to evaluate in field settings, and these findings could in turn have important implications for transmission modeling.

### Stool Shedding

Knowledge of the pattern and extent of stool shedding, both during acute infection and chronic carriage, was found to be an important factor when modeling transmission dynamics for *Salmonella* Typhi in Southeast Asia [24]. Prolonged stool shedding likely increases transmission, particularly in regions with poor sanitation [25]. Collection of daily stool samples during the acute challenge phase and clearance stools after completion of antibiotics allows the opportunity to follow bacterial shedding from time of bacterial ingestion to disease convalescence and bacterial clearance.

Data from the original Maryland challenge studies reveal a complex picture of stool shedding during the early phase of infection [26]. A proportion of participants who remained asymptomatic were noted to excrete *Salmonella* Typhi in their stools for several weeks. The frequency of excretion peaked at 2 weeks after challenge in both those who did and those who did not develop typhoid disease, with stool cultures often remaining positive for 3 weeks, after which the frequency of positive stool cultures declined, such that all participants had negative stool cultures by 6 weeks after challenge.

Whereas data on shedding during acute infection can be closely monitored, the Oxford typhoid challenge model offers limited scope to investigate shedding during chronic carriage as the latter is prevented by effective antibiotic therapy. Samples are collected a minimum of 2 weeks after completion of antibiotics to assess for chronic carriage and long-term shedding. In addition to this, participants with a history of gallbladder disease or ultrasonographic evidence of gallstones are excluded prior to study enrollment. To date, there has been no evidence of long-term *Salmonella* Typhi shedding in any challenged participant.

### Diagnostics

Human challenge models provide a controlled environment to develop and test the sensitivity of new diagnostics assays. For example, use of a novel culture polymerase chain reaction (PCR) assay targeting the fliC-d gene of *Salmonella* Typhi has resulted in the detection of asymptomatic primary *Salmonella* Typhi DNAemia from as little as 12 hours after challenge. Culture PCR was positive within 5 days of challenge in 10 of 40 participants, all of whom had negative routine blood culture during this period; 7 of 10 subsequently developed typhoid infection [27, 28]. Published diagnostic methods including antibody-in-lymphocyte supernatant [29] and *Salmonella* Typhi–specific anti-LPS salivary immunoglobulin A responses [30, 31] have been assessed using the typhoid challenge model and have produced variable results (unpublished data). These differences in immunological responses may be due in part to the short duration of illness and rapid commencement of antibiotic treatment required in the challenge model.

Natural infection with *Salmonella* Typhi is associated with antibody responses to a broad range of antigens including outer membrane (eg, OmpC or OmpS) or secreted proteins (eg, HlyE, CdtB) [32, 33]. Human challenge studies can aid in the identification of novel antigens for serological surveillance. The use of high-throughput antigen microarrays may lead to the identification of serodiagnostic and seroprotective antigens, which could be used to develop new rapid diagnostic assays or novel vaccines [34]. Long-term follow-up of participants from challenge studies offers the opportunity to assess for the persistence of antibody after infection, which may be difficult to achieve in field settings and knowledge of which will be key for serosurveillance and serodiagnosis studies.

### Assessing Vaccine Efficacy

Vaccines, together with other public health interventions, will play an important role in reducing the burden of disease caused by human-restricted typhoidal *Salmonella*. Currently licensed typhoid vaccines, parenteral Vi polysaccharide and oral live attenuated Ty21a, have several limitations including moderate vaccine efficacy and contraindication for use in young children. Human challenge studies provide the opportunity to assess novel vaccine candidates in relation to vaccine efficacy and immunogenicity prior to assessment in costly field trials.

Proof-of-principle of this approach was provided by the Maryland studies where the attenuated strain Ty21a was shown to protect volunteers challenged with *Salmonella* Typhi [14]. Our group has also utilized the model to test novel vaccine candidates. Recently we have investigated the protective efficacy of M01ZH09, a novel, single-dose, oral live attenuated vaccine [35], and Ty21a compared to placebo using the challenge model (ClinicalTrials.gov identifier NCT01405521), providing novel data on the potential for this vaccine to protect against typhoid infection and data relating to correlates of protection. The protective efficacy of a new Vi tetanus toxoid conjugate vaccine, Typbar-TCV (Bharat Biotech International Ltd), will also be assessed using the typhoid challenge model (ClinicalTrials.gov identifier NCT02324751).

## CONDUCTING HUMAN CHALLENGE STUDIES IN ENDEMIC REGIONS

Findings from challenge studies in a highly selected group of individuals in the United Kingdom may not directly correlate with those found in endemic regions. The response to enteric pathogens or oral vaccines, for example, may differ in an African setting due to preexisting natural immunity, genetic differences, or comorbid conditions [19, 36]. Malaria challenge studies have been conducted in African sites for several years in an attempt to account for some of these variables following malaria challenge [37]. Furthermore, there is precedent for undertaking human challenge studies with enteric pathogens in resource-limited settings [38, 39]. Although undertaking a typhoid (or NTS) challenge study in Africa would require careful study design, detailed discussions between key stakeholders, and significant financial investment, such studies may offer a means to study disease in the population of interest. This would allow further investigation into the impact variables such as previous *Salmonella* exposure, dietary factors, intestinal microbiota, and the genetic profile of the host population, have on disease pathogenesis and host immune responses [40].

## DEVELOPING AN NTS HUMAN CHALLENGE MODEL

Given the increasing role of NTS strains in causing invasive *Salmonella* disease in Africa, in addition to the scientific and translational benefits provided by established human enteric fever challenge models, increasing attention has been given to the feasibility of developing an NTS human challenge model [18]. A controlled setting to study host–pathogen interactions, bacterial dynamics, and assess NTS vaccine candidates would offer a novel approach and a rapid way to advance our understanding of NTS disease. Important considerations in the development of an NTS human challenge model would include identification of an appropriate starting dose of challenge agent, development of a suitable dose escalation strategy, and determining the need for bicarbonate pretreatment to neutralize stomach acid prior to challenge [41], in addition to the issues discussed below.

### Strain Selection and Manufacture

Strain choice is integral to the design of a human challenge study. Ingestion of the challenge strain needs to result in the required disease endpoint, which is generally observable, reproducible clinical infection. The early Maryland studies, for example, failed to induce enteric fever after challenge with a variety of deposited Ty2 strains [42], but successfully reproduced features of bacteremia and clinical symptomatology following challenge with the Quailes strain of *Salmonella* Typhi—a Vi-expressing strain isolated from a then-recently diagnosed chronic carrier (Mrs Quailes) [22, 43]. Challenge strains should also express the required virulence characteristics and share features consistent with other circulating strains, which will allow study findings to be applied to contemporary field settings. Undertaking a challenge study with an H58 isolate of *Salmonella* Typhi, for example, would offer an opportunity to understand host–pathogen interactions using a strain of contemporary significance. Based upon current knowledge of NTS serovars and strains, *Salmonella* Typhimurium ST313 would be an appropriate choice to develop as a challenge agent, as it accounts for much of the iNTS disease seen in sub-Saharan Africa and, similar to *Salmonella* Typhi, appears to display features of human adaptation. In a similar fashion to the development of the *Salmonella* Paratyphi challenge strain (NVGH308), an isolate obtained from the blood culture of a clinical case of paratyphoid infection in Kathmandu, Nepal (NCT02100397), an ST313 isolate from the field could, in theory, also be isolated and manufactured to Good Manufacturing Practice standards for use in challenge studies. A series of quality control tests are performed before the strain can be used in human challenge studies including antimicrobial susceptibility and stability testing. In some settings, the substantial cost of strain manufacture may represent a major barrier to the development of an NTS challenge strain and, as such, significant financial investment and collaboration by funders and/or industrial partners would be required.

### Endpoint Definition

In enteric fever challenge studies, fever and/or bacteremia have been used as objective measurable endpoints of infection. Defining an endpoint after NTS challenge that is both safe and clinically meaningful is particularly difficult. Fever or bacteremia would represent the most clinically relevant features of iNTS disease; however, it is uncertain if healthy, immunocompetent volunteers would develop inflammatory gastroenteritis or invasive disease after challenge. Even in the African setting, invasive disease is uncommon in healthy adults. Development of bacteremia may be associated with a high risk of complications such as endovascular infection, meningitis, or septic shock. Alternative endpoints could include diarrhea, isolation of challenge agent from stool, or a predetermined duration of stool shedding. Such endpoints have been used in other enteric challenge studies and would be associated with less risk to study participants. Although such a model would not address potentially important factors related to invasive disease, such as systemic host–pathogen interactions, it would assist with the study of mucosal immunity, stool shedding, and the effect NTS vaccine candidates have on preventing gastroenteritis and bacterial shedding, and as such, may have implications on preventing NTS transmission in the field.

### Safety

The overall mortality rate of iNTS disease is estimated at 20% in the African setting [8] and may be much higher in particular at-risk groups [44]. The mortality rate of iNTS in high-income countries, whereas lower than that seen in African settings, may be as high as 5% [45]. The potential for serious complications including septic shock, endovascular infection, or meningitis would be a potential concern if using systemic infection as an endpoint [46], and consideration should be given to screen participants for potential predisposing conditions, such as aneurysmal arterial disease or valvular heart disease. An inpatient NTS challenge model could reduce the risk to participants by allowing close clinical monitoring, cautious dose escalation, and prompt antibiotic treatment as well as minimizing the risk of transmission to close household contacts [47]. However, an inpatient model would require dedicated facilities and staff, with substantial attendant costs. Detailed discussion and agreement between a range of key stakeholders, including the study team, sponsors, public health services, regulatory agencies, and ethics committees, would be required to minimize risk when designing an NTS challenge model [17, 18].

## CONCLUSIONS

Despite growing evidence demonstrating the significant morbidity and mortality caused by invasive *Salmonella* disease in Africa, many factors relating to disease transmission remain unknown. Human challenge studies can shed light on several of these unknown areas including strain infectivity, duration of stool shedding, and detection of early and late disease markers. Use of typhoid challenge models also provides a platform to evaluate novel vaccine candidates, which could play a key role in future disease control efforts given increasing rates of antimicrobial resistance among *Salmonella* Typhi isolates from Africa. We have shown that *Salmonella* Typhi human challenge models can be performed safely and offer a unique means of studying disease from the point of infection through to diagnosis, treatment, and convalescence. An NTS human challenge model could offer similar advantages of obtaining valuable human-specific data for a currently neglected and serious disease, but several knowledge gaps would have to be filled before such a model could be seriously contemplated. While a number of well-described risk factors for iNTS have been identified, invasive disease is a relatively infrequent occurrence in the African setting following exposure to NTS serovars in the gastrointestinal tract. Human challenge studies could allow further exploration of the host and pathogen factors associated with the iNTS phenotype; however, the unpredictable response to a controlled challenge in healthy volunteers is a potential hurdle. The low attack rate in the general population may preclude the successful development of a model, even at high challenge dose. On the other hand, there is a real potential for serious complications with iNTS. Careful consideration of ways to mitigate the risk to study participants may preclude development of a human challenge model in the short term, but it is hoped that insights from enteric fever challenge studies can benefit ongoing disease surveillance and contribute to the collaborative effort to control invasive *Salmonella* disease across Africa.
